# Visual Perception and Visuomotor Reaction Speed Are Independent of the Individual Alpha Frequency

**DOI:** 10.3389/fnins.2021.620266

**Published:** 2021-04-08

**Authors:** Thorben Hülsdünker, Andreas Mierau

**Affiliations:** ^1^Department of Exercise and Sport Science, LUNEX International University of Health, Exercise and Sports, Differdange, Luxembourg; ^2^Institute of Movement and Neurosciences, German Sport University Cologne, Cologne, Germany

**Keywords:** sport, brain, reaction time, athlete, EEG, event-related potential, behavior, performance

## Abstract

While the resting-state individual alpha frequency (IAF) is related to the cognitive performance and temporal resolution of visual perception, it remains unclear how it affects the neural correlates of visual perception and reaction processes. This study aimed to unravel the relation between IAF, visual perception, and visuomotor reaction time. One hundred forty-eight (148) participants (28 non-athletes, 39 table tennis players, and 81 badminton players) investigated in three previous studies were considered. During a visuomotor reaction task, the visuomotor reaction time (VMRT) and EMG onset were determined. In addition, a 64-channel EEG system identified the N2, N2-r, and BA6 negativity potentials representing the visual and motor processes related to visuomotor reactions. Resting-state individual alpha frequency (IAF) in visual and motor regions was compared based on sport experience (athletes vs. non-athletes), discipline (badminton vs. table tennis), and reaction performance (fast vs. medium vs. slow reaction time). Further, the differences in the IAF were determined in relation to the speed of neural visual (high vs. medium vs. low N2/N2-r latency) and motor (high vs. medium vs. low BA6 negativity latency). Group comparisons did not reveal any difference in the IAF between athletes and non-athletes (*p* = 0.352, η_*p*_^2^ = 0.02) or badminton and table tennis players (*p* = 0.221, η_*p*_^2^ = 0.02). Similarly, classification based on the behavioral or neural performance indicators did not reveal any effects on the IAF (*p* ≥ 0.158, η_*p*_^2^ ≤ 0.027). IAF was not correlated to any of the behavioral or neural parameters (*r* ≤ 0.10, *p* ≥ 0.221). In contrast to behavioral results on cognitive performance and visual temporal resolution, the resting state IAF seemed unrelated to the visual perception and visuomotor reaction speed in simple reaction tasks. Considering the previous results on the correlations between the IAF, cognitive abilities, and temporal sampling of visual information, the results suggest that a higher IAF may facilitate the amount and frequency but not the speed of information transfer.

## Introduction

In the human brain, oscillatory activity in the alpha rhythm of around 10 Hz is the dominant frequency. The alpha frequency band includes frequencies between 7.5 to 12 Hz (Klimesch, 1999) and is most pronounced in the parieto-occipital regions during eyes closed resting state conditions. Since the development of perceptual, cognitive, and motor expertise is accompanied by modulations in the alpha rhythm (Babiloni et al., 2008; Park et al., 2015), Cheron et al. (2016) proposed the alpha rhythm as one of five cortical biomarkers for sport performance. While previous studies focused on the effects of the amplitude of alpha oscillations on behavioral performance, there is an increasing research in healthy populations or patients investigating the peak of alpha activity within the alpha frequency band, the so-called individual alpha frequency (IAF) (Bazanova and Vernon, 2014). The IAF is usually defined as the individual spectral peak in the alpha band [individual alpha peak frequency (iAPF)] or the center of gravity (COG) (Klimesch et al., 1993).

The individual alpha frequency measured during resting state conditions has repeatedly been shown to be related to cognitive performance. Specifically, the IAF was positively correlated to performance in tasks on memory (Klimesch et al., 1993), language comprehension (Bornkessel et al., 2004), and processing speed (Grandy et al., 2013). Furthermore, Jin et al. (2006) observed a negative relation between IAF and conflict choice reaction time indicating higher alpha frequencies speed up decision making processes. In a study by Richard Clark et al. (2004), IAF values were further related to superior working memory performance in a reverse digit span task. Younger participants with a higher IAF outperformed older adults with lower peak alpha frequency values. Interestingly, age-related slowing of alpha frequencies was most pronounced in the frontal regions and only the frontal IAF predicted the working memory task performance (Richard Clark et al., 2004). Since the frontal brain areas are involved in working memory processes, these findings emphasize the assessment of the individual alpha frequency in task-related cortical areas.

The abovementioned cognitive abilities are well established to be associated with sport performance. Scharfen and Memmert (2019) reported a direct relation between working memory capacity and soccer-specific motor performance such as dribbling or ball juggling in youth athletes. Moreover, baseball players have previously been shown to exhibit superior decision-making abilities that in turn were associated with an earlier and more pronounced activation in the EEG (Muraskin et al., 2015). Accordingly, experts performing on a high-performance level may be characterized by a higher IAF. However, although Cheron et al. (2016) proposed the alpha frequency as a cortical biomarker of performance, research on the IAF in athletes is limited. Currently, the study of Christie et al. (2017) is the only one addressing a relation between the athlete’s IAF and sport performance. While the differences between athletes on different performance levels did not reach significance, the authors emphasized the need for further research addressing the IAF in groups of greater diversity such as athletes and non-athletes. Moreover, while previous research focused on behavioral outcomes, there is a lack of studies addressing the effects of IAF on neural correlates of behavioral performance. Therefore, this study aims to identify differences in the IAF between high-level youth and adult athletes and non-athletes and evaluate the effects of IAF on neural signal processing and behavioral performance.

Athletes, especially those participating in ball and racquet sports, develop exceptional perceptual and perceptual-motor abilities in the visual system which is reflected by a faster visuomotor reaction times when compared to non-athletes (Zwierko et al., 2010; Hülsdünker et al., 2018a). Moreover, the athlete’s superior reaction speed is accompanied by characteristic changes in cortical activation (Hülsdünker et al., 2018a) making visuomotor reaction tasks a promising model for studying the effects of IAF on the neural processing and behavioral performance. Previous research addressing the neural basis of faster visuomotor reactions in badminton athletes revealed an earlier activation of cortical regions corresponding to the motion-sensitive area MT in athletes when compared to non-athletes (Hülsdünker et al., 2017b). Importantly, the latency of the N2 and N2-r motion onset visual evoked potentials in the EEG did not only contribute to the athletes’ faster reaction speed when compared to non-athletes but also explained 60–80% of the variance in reaction time between athletes in both badminton and table tennis (Hülsdünker et al., 2017a, 2019).

The N2 is a stimulus-locked cortical potential observed around 150–250 ms following a motion stimulus onset. It is located in the visual motion sensitive area MT (Henning et al., 2005) and is suggested to be associated with the perception of visual motion information (Henning et al., 2005; Kuba et al., 2007; Hülsdünker et al., 2019). Accordingly, interfering with signal processing in area MT in this interval using TMS has repeatedly been shown to significantly delay visuomotor reactions (Laycock et al., 2007; Bosco et al., 2008). Importantly, activation in area MT is not only a stimulus- but also response-locked to the onset of a motor response (Kawano et al., 1994; Hülsdünker et al., 2017b, 2019). This response-locked N2 potential (N2-r potential) occurs around the initiation of the motor response. It is strongly related to the visuomotor reaction speed and suggested to reflect the processing of visual information (Hülsdünker et al., 2019).

In addition to the two visual potentials, event-related activity was also observed over pre-and supplementary motor regions (Laycock et al., 2007; Hülsdünker et al., 2018a). This negative and only stimulus-locked potential occurs 150–200 following the stimulus onset. Since TMS stimulation at that interval revealed a substantial delay of reaction speed (Schluter et al., 1999), this so-called BA6 negativity potential was previously suggested to reflect the transfer of visual information into a motor response (Ledberg et al., 2007; Hülsdünker et al., 2017b, 2019). However, although athletes were characterized by a faster activation of motor areas as reflected by a lower BA6 negativity latency, this did not substantially contribute to the speed of visuomotor reactions. The MNI coordinates for the N2/N2-r potentials and the BA6 negativity potential derived from inverse localization analyses (Hülsdünker et al., 2017a, b, 2019, 2020) were well in accordance with fMRI research on the location of area MT (Henning et al., 2005; Kolster et al., 2010) and a meta-analysis on the location of the pre- and supplementary motor cortex (BA6) (Mayka et al., 2006), respectively.

The combined pattern of results raises the question if similar to cognitive abilities, superior perceptual and perceptual-motor processes may be related to the IAF. If yes, this should be reflected by a higher IAF in athletes when compared to non-athletes and a negative correlation between the IAF and reaction time.

A possible relation between the IAF and visuomotor performance is supported by previous research emphasizing the dependence of visual perception and processing on the alpha rhythm. Specifically, the integration of visual information is suggested to be framed by perceptual cycles that depend on the frequency of alpha oscillations (VanRullen, 2016). On the behavioral level, this is reflected by a higher temporal resolution of visual perception with an increasing IAF that in turn manifests in a more accurate discrimination of single flashes in a double-flash stimulation paradigm (Samaha and Postle, 2015). Similarly, Cecere et al. (2015) reported a direct relation between the IAF and the temporal window size for the integration of perceptual information. In an audio-visual experiment, participants were exposed to a double-beep presentation accompanied by a visual stimulation on the first beep to induce a double flash illusion. The authors reported a correlation between the participant’s IAF and the window size for the double-flash illusion. This was further supported by occipital transcranial alternating current stimulation (tACS) at frequencies below and above the participant’s IAF which resulted in a frequency dependent change of the temporal window size in which an illusion occurred.

These findings suggest that a higher IAF facilitates the amount and temporal resolution of information transfer which can explain the superior visual (Cecere et al., 2015; Samaha and Postle, 2015) and cognitive performance (Grandy et al., 2013). Similarly, the IAF-dependent integration of visual information may also affect the visual perception and visuomotor reaction speed. Specifically, research on both animals (Cook and Maunsell, 2002) and humans (Stevens et al., 2009) suggest that visual motion perception in area MT is encoded based on the successive integration of visual information over frames until a threshold for motion perception is reached. Such a threshold model is further supported by our previous research where the response-locked N2 (N2-r) peak was observed after the EMG onset. This indicates that the initial perception of visual motion does not necessarily coincide with the peak of the N2 potential but may occur earlier (Hülsdünker et al., 2017b, 2019, 2020). A higher IAF accompanied by a higher rate of visual information transfer may allow reaching this threshold faster which would result in a lower N2 latency and faster visuomotor reaction speed. Another line of argument supporting a relation between IAF, visual perception, and visuomotor reaction speed is the temporal profile of developmental changes in N2 latency, visuomotor reaction time, and IAF that are of high similarity. Kuba et al. (2007) have shown reductions in N2 latency during development until the age of about 18 years followed by a steady increase during further aging. Similarly, the visuomotor reaction speed accelerates during maturation (Miyaguchi et al., 2013; Scantlebury et al., 2014) before it slows down after the age of about 20 (Fozard et al., 1994). A similar time course is also observed for the IAF that increases until a peak of 9.5–11 Hz around the age of 20 before it starts slowing after about 40 years of age (Klimesch, 1999). Scantlebury et al. (2014) also reported a direct relation between improvements in the reaction speed and changes in the white matter structure. Similar results have been reported for the IAF that is correlated to white matter integrity (Valdés-Hernández et al., 2010). These findings may suggest that expert athletes who have developed exceptional visual (N2/N2-r latency) and visuomotor (reaction time) performance should be characterized by a higher IAF that allows rapid visual perception and processing. Furthermore, given the direct relation between N2/N2-r latency and visuomotor rection speed, this relation may also be observed between the N2/N2-r and the IAF.

However, although the abovementioned findings support a potential relation between the IAF, neural correlates of visual perception (N2/N2-r), and behavioral performance (reaction speed), this has not been investigated yet. Moreover, while previous research focused on the relation between the IAF and behavioral performance, this study is the first evaluating the link between the IAF, neural signal processing, and behavioral performance.

To determine the effect of individual alpha frequency on visual perception and visuomotor reaction speed, this study included a large sample of 148 participants (120 athletes, 28 non-athletes) that were tested in different experiments over a period of 5 years. All participants performed a resting state measurement as well as a visuomotor reaction task. Resting-state IAF was determined in visual and motor regions corresponding to the N2, N2-r, and BA6 negativity potentials. Furthermore, the onset of muscular activation (EMG onset) and visuomotor reaction time (VMRT) as behavioral correlates of reaction performance were measured. Group comparisons examined differences in the IAF related to sport experience (athletes vs. non-athletes) and discipline (table tennis vs. badminton). Supplementary classification analyses investigated the effects of reaction skill level (slow vs. medium vs. fast responders) and neural activation speed (slow vs. medium vs. fast activation) on the IAF. In addition, correlation and regression analyses quantified the relation between the IAF, reaction speed, and neural activation latency.

Based on the abovementioned parallels in the development of IAF, N2 latency, and visuomotor reaction time (Fozard et al., 1994; Klimesch, 1999; Scantlebury et al., 2014), we expect a direct relation between IAF, N2/N2-r latency, and reaction speed. This is further supported by the findings of Jin et al. (2006). Although the authors did not report a significant correlation between the IAF and simple reaction time, they still observed a medium effect size of *r* = 0.47. Since 37 participants would be required to identify a significant relation between the IAF and simple reaction time with a power of 0.85 (see also sample size calculation), the authors may have missed a significant relation due to the small sample size (*n* = 14) and, thus, a comparatively low test power (power = 0.41). In terms of group comparisons, we expect a higher IAF in athletes when compared to non-athletes and fast when compared to slow responders. A higher IAF may allow faster accumulation of motion information and thus contribute to an earlier visual perception (N2/N2-r) and visuomotor reaction speed. Since the BA6 negativity latency was not related to the visuomotor reaction speed in athletes (Hülsdünker et al., 2017a, 2019), there should also be no relation to IAF.

## Methods

### Sample Size Calculation

Sample size calculations were performed in the G^∗^Power (3.1.9.6) software package (Faul et al., 2007, 2009). Since no effect sizes were available for the relation between IAF and perceptual and reaction parameters, we used the results of Grandy et al. (2013) as a reference. The authors reported correlation coefficients between IAF and cognitive abilities (speed, memory, reasoning) between *r* = 0.25 and *r* = 0.37 for young participants (25.5 years). The correlation of *r* = 0.31 for the “speed” parameter was used for the sample size calculation since the cognitive speed ability should be closest to the performance outcome variables of this study. Using a bivariate normal model with an alpha level of 0.05 and a test power of 0.85 in a two-tailed statistic, sample size calculation yielded a required sample of 90 participants. Jin et al., 2006 reported a relation between simple reaction time and alpha peak frequency (calculated differently when compared to this study) of 0.47. With a similar alpha level and test power, this would result in a required sample of 37 participants.

Since we followed the performance classification approach of Christie et al. (2017) for supplementary comparisons between IAF and visuomotor reaction performance, this study was used as an orientation for sample size calculation related to the group comparisons. Although effect sizes were not reported, sample size was calculated based on the group mean and pooled standard deviation. Assuming an alpha level of 0.05 and a power of 0.85, the calculation resulted in a sample of 31 participants per group when subdividing into three groups (terciles) in a one-way ANOVA statistic with GROUP as the between-subject factor. With a sample of 148 participants and an expected higher effect size due to greater differences in the behavioral performance between subjects when compared to the study of Christie et al. (2017), the sample size was considered sufficient to investigate the effects of interest.

### Participants

Data from 148 participants were considered for this study. Participants were adult and young badminton players, young table tennis athletes, as well as adult non-athletes. Only athletes with an experience of at least 4 years in their sport and a minimum of 8 h of training per week were included. Furthermore, all badminton and table tennis players had to perform at the highest level in their respective age group and participate in national and international competitions. Participants were excluded if they suffered from epilepsy, migraine, or other neurophysiological disorders or had a visual acuity less than 20/20. Furthermore, participants had to confirm that they were free of injury, had no pain or other kind of discomfort, and experience no limitation during their daily routines and exercise. Participants were provided with the experimental protocol and their written consent was obtained. In case the participants were under the legal age, the consent form was also signed by the parents or a guardian. All experiments were approved by the local research ethics committee in accordance with the declaration of Helsinki. The group characteristics are presented in Table 1. Due to a missing alpha peak as identified by visual inspection, three participants were excluded from the data analysis.

**TABLE 1:** Summary of participant data.

	Group	Adult badminton	Adult non-athletes	Young table tennis	Young badminton
Participant data	Sample size (*n*)	36 (11 m, 25f)	28 (11 m, 17f)	39 (19 m, 20f)	45 (29 m, 16f)
	Age (years)	19.8 (±3.9)	18.7 (±2.6)	13.5 (±1.5)	13.7 (±1.4)
	Height (cm)	174.7 (±8.8)	173.6 (±9.6)	162 (±9.7)	166 (±11.4)
	Weight (kg)	67.0 (±8.5)	65.2 (±14.89)	51.5 (±10.6)	53.8 (±11.0)
	Training experience (years)	11.0 (±3.9)		7.0 (±1.4)	6.3 (±2.2)
	Training load (h/week)	15.2 (±5.3)		19.0 (±5.9)	12.4 (±3.7)
	Handedness	33 right, 3 left	23 right, 5 left	31 right, 8 left	39 right, 6 left
Experiment data	Experiment time (hh:mm)	12:49 (±02:48)	14:00 (±03:30)	14:36 (±03:10)	14:52 (±02:48)

*Values are presented as mean (±standard deviation). Since non-athletes do not have training experience and a weekly training load, these fields are kept empty.*

### Experimental Protocol

All participants performed two resting-state EEG measurements and a visuomotor reaction task. Across studies, a simple reaction task has been used to focus on visual perception performance and avoid the interference of cognitive processes. A Landolt test confirmed that the participants had a visual acuity of at least 20/20. All tests were performed on a computer screen.

#### Resting State EEG

Prior to the experiment, resting state EEG was measured for 1 min with the eyes closed. Athletes were seated in a height-adjustable chair with their head placed on a chinrest to ensure eyes were level with the center of the screen. During the reaction task, participants had to focus on a red fixation point at screen center. Earplugs were used to avoid distraction from environmental noise. For the resting state measurements a text was shown on the screen informing participants to close their eyes. Three seconds after text presentation, the 60-second resting state measurement was started. The end of the rest interval was indicated by an auditory beep cue (1,000 Hz). The same procedure was used for the resting state measurement after the experiment.

#### Visuomotor Reaction Tasks

Although protocols were slightly different between studies, all experiments had a length between 31 and 33 min and included a visuomotor reaction task in response to a 5 Hz motion onset stimulus. This condition is used for data analysis in this study.

Details on the stimulation can be found elsewhere (Hülsdünker et al., 2017a, 2019). In short, the visual motion sensitive area MT was activated by radial motion onset stimuli presented at a mean luminance of 17 cd × m^–2^ and a Michelson contrast of 10%. A gray circle (17 cd × m^–2^) was in the middle of the screen (central 5°). The viewing distance was 500 mm resulting in a visual field of 44.2 × 33.8°. The visual motion stimulus had a duration of 200 ms with an interstimulus interval varying between 2 to 6 s (average = 4 s) to avoid temporal anticipation. Stimuli were randomly either contracting or expanding to reduce adaptation effects. Participants were instructed to push a button on a response pad with the index finger of their dominant hand whenever they perceived a motion onset on the screen. Dependent on the study, the number of reaction trials varied between 60 and 80.

All stimuli were programmed using the CRS toolbox (Cambridge Research Systems, Rochester, United Kingdom) implemented in the Matlab (The Mathworks Inc., Natick, MA, United States). A ViSaGe MKII (Cambridge Research Systems) visual stimulus generator was used for stimulation. Stimuli were presented on a 22″ HP1230 color CRT screen with a refresh rate of 120 Hz and a resolution of 1,024 × 768 pixels. Prior to each participant, the screen luminance was calibrated using a ColorCalII colorimeter (Cambridge Research Systems). Frame-synchronously with the visual stimulation, electrical trigger pulses were sent by the ViSaGe to synchronize all recording devices.

#### Data Acquisition

EEG was acquired using a 64-channel actiChamp amplifier (Brain Products GmbH, Munich, Germany). Electrodes were equally distributed over both hemispheres based on the 10:10 system (Jurcak et al., 2007). One electrode was used to measure the electrooculographic activity. Ground and reference electrodes were placed on the positions Afz and FCz, respectively. Impedance was kept below 15 kΩ. Data was sampled at 1,000 Hz and online low-pass filtered at 280 Hz. EMG was measured on the flexor digitorum superficialis muscle of the participant’s dominant hand. A double differential electrode connected to a Bagnoli amplifier (Delsys, Natick, MA, United States) recorded at a sampling rate of 1,000 Hz and an online band-pass filter between 20–450 Hz. The speed of visuomotor reactions was determined with a Cedrus RB-530 or RB-844 response pad (Cedrus Cooperation, San Pedro, CA, United States). Similar to the EEG and EMG, the sampling frequency was 1,000 Hz. EEG, EMG, and response pad were synchronized by the ViSaGe electrical trigger pulses.

#### Data Analysis

##### Individual alpha frequency definition

For the identification of the IAF, resting state EEG data was band-pass filtered [0.3–35 Hz, IIR zero phase-shift butterworth filter (order = 8), notch filter at 50 Hz], and segmented into epochs of 4 s length (2 s overlap). A semiautomatic artifact rejection procedure (maximal voltage step: 50 μV, maximal amplitude within 200 ms: 100 μV) followed by a visual signal inspection excluded artefactual segments. Similar to our previous studies, the current source density (spline order: 4, Legendre of Polynomials: 10, Lambda: 1e^–15^) was calculated to differentiate between focal alpha rhythms. Single trial epochs were Fourier transformed (frequency resolution 0.244 Hz, frequency precision = 0.25 Hz, hanning window: 10%) and averaged across segments. Individual alpha frequency was determined for visual (primary visual cortex and area MT) and motor regions [pre- and supplementary motor cortex (BA6)] that has previously been identified to differentiate athletes from non-athletes (Hülsdünker et al., 2016, 2017b) and show a strong relation to the speed of visuomotor reactions (Hülsdünker et al., 2017a).

To determine the IAF in the three regions of interest, data was analyzed on the channel level. However, according to previous studies and based on the cortical representation of EEG sensors determined by Koessler et al. (2009), the selected electrodes were representative for the area MT, the primary visual cortex (V1), and the pre-and supplementary motor area (BA6). This is further supported by the location of the N2, N2-r, and BA6 negativity potentials derived from inverse localizations (see below). Bilateral activation corresponding to area MT was defined as the average frequency spectrum of electrode positions PO7, P7, P5, PO8, P8, and P6. BA6 activity was reflected by electrode positions FC1, FCz, and FC2 while electrode position Oz corresponded to the primary visual cortex (V1). The definition of regions of interest followed the procedure of our previous studies and allowed a direct comparison between IAF and cortical event-related potentials derived from the visuomotor reaction task.

Due to possible multiple peaks in the alpha frequency spectrum (Klimesch, 1999), the center of gravity frequency (COG) rather than the individual alpha peak frequency (iAPF) was calculated. Chiang et al. (2011) observed multiple alpha rhythms in about 44% of the participants in a large sample of about 1,500 subjects aged between 6 and 86 years. Based on the suggestions of Haegens et al. (2014) and Christie et al. (2017) emphasizing a wide alpha frequency band, the alpha band was defined between 7 and 14. The IAF, determined based on the center of gravity (COG) frequency, was calculated for MT, BA6, and V1 according to equation 1, as proposed by Klimesch (1999).

(1) $$\frac{\sum \left(a\left(f\right)\times f\right)}{\left(\sum a\left(f\right)\right)}$$

where *a* represents the power spectral estimate at a frequency (*f*).

##### Event-related cortical potentials

The definition of event-related potentials in visual and motor regions has been described in detail in our previous studies (Hülsdünker et al., 2017b, 2019). In short, following semiautomatic artifact rejection and correction, respectively, an extended runica algorithm was applied to each dataset using the EEGlab software package in Matlab (Delorme and Makeig, 2004). In a semi-automatic approach, artefactual components were first identified with the SASICA procedure (Chaumon et al., 2015) and subsequently visually checked based on time course, mapping, and frequency spectrum. For experiments with 60 and 80 stimuli, on average, 48 (±6.7) and 71.4 (±5.6) segments were considered for data analysis, respectively. Across participants, the ICA removed 7.7 (±2.8) components from the data. After the ICA back-transform, the EEG channel data was transformed using the CSD.

Since a full-field visual stimulation was used, the bilateral N2 component of the motion onset visual evoked potential was identified based on the average activity of sensors PO7, P7, P5, PO8, P8, and P6 that best represent area MT (Koessler et al., 2009). The same sensors have been used in a series of previous experiments and have been shown to validly reflect a negative potential that was located in cortical areas corresponding to area MT (Henning et al., 2005) when using LORETA inverse localization (Hülsdünker et al., 2017b, 2019, 2020). The activity in this newly generated, pooled channel was then averaged across single trial segments. The N2 was determined as the maximum negative peak between 100–300 ms after motion stimulus onset. In the same interval, the BA6 negativity potential was determined as the maximum negative peak in the signal averaged across electrode positions FC1, FCz, and FC2. These sensors have previously been shown to correspond to the pre-and supplementary motor cortex in 100% of the participants (Koessler et al., 2009).

In addition to stimulus-locked cortical activity, the response-locked N2-r component in the electrodes corresponding to area MT (PO7, P7, P5, PO8, P8, and P6) was identified. To this end, artifact free epochs were first re-segmented based on the EMG onset (−500–200 ms) and the average of single-trial segments was calculated. The N2-r potential was then identified as the maximum negative peak between −50–50 ms relative to the EMG onset.

**FIGURE 1:**
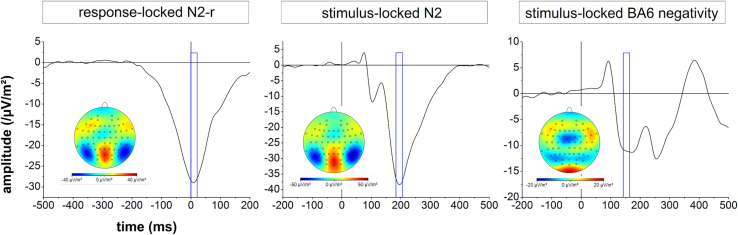
Temporal profile and cortical mapping of the event-related potentials (ERPs) of interest (response-locked N2 = left figure, stimulus-locked N2 = middle figure, stimulus-locked BA6 negativity = right figure). The blue box reflects a time window of 10 ms for the calculation of the cortical mappings. Please note that the different scaling for the mappings is due to variations in ERP amplitude.

For each participant, the N2, N2-r, and BA6 negativity potentials were automatically detected in the time window of interest. However, the latencies of all ERP components were checked for each participant individually and in case of uncertainty, cortical activity mappings and single channel time courses were considered to estimate the correct timing. The time courses and mappings associated with the N2, N2-r, and BA6 negativity potentials across all participants are presented in Figure 1.

##### Visuomotor reaction speed

The speed of visuomotor reactions was defined as the time between stimulus presentation and initial muscular activation (EMG onset) as well as button press [visuomotor reaction time (VMRT)]. Trials with reaction times faster than 100 ms or slower than 500 ms were excluded. For EMG onset definition, EMG data was first baseline-corrected (−500–0 ms) for each trial. Based on the procedure of Hodges and Bui (1996), the EMG onset was then defined as the point where the low-pass filtered (50 Hz) and smoothed (25 ms moving average) signal exceeds three standard deviations from baseline (−500–0 ms). Afterward, each EMG onset timing was visually checked.

### Statistics

All statistical analyses were performed in Statistica 7.1 (Statsoft, Tulsa, OK, United States) and SPSS 27 (IBM, Armonk, NY, United States). Normal distribution was checked and confirmed for all parameters using the Kolmogorov-Smirnoff test (*p* > 0.068).

#### Control Analyses

This experiment aimed to identify the effects of sport expertise, type of sport, and visuomotor reaction performance on the IAF. However, since there may have been other factors influencing the IAF, a series of control analyses was performed to identify potential covariates for subsequent group statistics, correlation and regression analyses.

In their experiment in ice-hockey players, Christie et al. (2017) reported a shift from the pre-to the post-test IAF. Therefore, to evaluate the stability of the IAF between pre- and post-experiment measurements, a repeated measures ANOVA with the between-subject factor GROUP (adult badminton, adult control young badminton, young table tennis) and the within-subject factors TIME (pre, post) and REGION (MT, V1, and BA6) was conducted. Results revealed a difference between the pre- and post-IAF in area MT, thus, the IAF change score in area MT was included as a covariate for group comparisons and a factor in partial correlation and regression analyses.

Previous research suggested a relation between the IAF and age (Klimesch, 1999). Furthermore, other influencing factors such as the time of the day the experiment was conducted, the athletes training experience and the weekly training load may be related to the IAF. In a first step, these parameters were included as predictors in a stepwise forward multiple regression to identify its impact on the IAF in cortical regions corresponding to area MT, BA6, and V1 that were considered as the dependent variables.

To further elaborate on a potential effect of these influencing factors, participants were assigned to quartiles based on their age, experiment participation (time of the day), training experience, and training load. Quartiles were used to contrast extremes in the parameters of interest. In ANOVA analyses, the first and fourth quartiles were compared with the between-subject factor GROUP (first quartile, fourth quartile) and the within-subject factor REGION (MT, V1, and BA6). To test for potential effects of gender, an ANOVA with GENDER as between-subject factor REGION (MT, BA6, and V1) as within-subject factor was conducted.

Significant correlations and differences between quartiles were only observed for age. The factor age was thus included as a covariate for all group comparisons, multiple regressions, and in partial correlation analyses. All other factors (experiment time, training experience, training load, and gender) did not reveal any effect on the IAF and were not considered as influencing factors. Differences between the 1st and 4th quartile for all classification variables are presented in Table 2.

**TABLE 2:** Group characteristics for group comparisons based on first and fourth quartile.

Control Parameter	1st quartile	4th quartile
Age (years)	12.3(±0.7)	20.2(±3.2)
Time (hh:mm)	9:48(±01:24)	17:42(±00:54)
Training experience (years)	4.6(±0.9)	11.2(±3.1)
Training load (h/week)	9.3(±1.4)	22.8(±4.6)

*Data represent mean (±standard deviation).*

#### Group Comparisons

Since visuomotor reaction speed is substantially faster in badminton players when compared to non-athletes (Hülsdünker et al., 2017b) and may also differentiate athletes participating in different sports, group comparisons were performed to compare the IAF between athletes and non-athletes as well as table tennis and badminton players. An ANCOVA with the between subject factor GROUP (adult badminton athletes, adult non-athletes), the within-subject factor REGION (MT, V1, and BA6), and age and pre-post change in IAF (area MT) as covariates was performed to identify differences in IAF, and reaction speed (EMG onset, VMRT) between athletes and non-athletes. A second ANCOVA with the between subject factor GROUP (young badminton, young table tennis), the within-subject factor REGION (MT, V1, and BA6), and the same covariates evaluated the effects of discipline on IAF.

#### Multiple Regression and Partial Correlation Analyses

To identify direct relations between the IAF, behavioral performance, and neural activation latency, multiple linear regression models and partial correlation analyses were conducted. For multiple linear regressions, the IAF in cortical areas corresponding to MT, V1, and BA6 was predicted by all behavioral (EMG onset, VMRT) and neural (N2, N2-r, and BA6 negativity) parameters. Partial correlation analyses were calculated between EMG onset/VMRT and IAF across participants as well as for the relation between IAF and neurophysiological parameters as reflected by the N2, N2-r, and BA6 negativity potentials. Age and pre-post change in MT IAF were included as influencing factors in all analyses.

Bonferroni correction was applied to *post-hoc* analyses to control for multiple comparisons. Effect sizes were considered small (η_*p*_^2^ = 0.01; *r* = 0.1; *d* = 0.2), medium (η_*p*_^2^ = 0.06; *r* = 0.3; *d* = 0.5), or large (η_*p*_^2^ = 0.14; *r* = 0.5; *d* = 0.8). Significance levels were defined as follows: ^∗^*P* < 0.05, ^∗∗^*P* < 0.01, and ^∗∗∗^*P* < 0.001.

#### Supplementary Performance Classification Analyses

Supplementary analyses using a classification approach to investigate the effects of visuomotor reaction time and neural activation speed on the IAF can be found in the Supplementary Results methods and results section.

## Results

### Control Analyses

**FIGURE 2:**
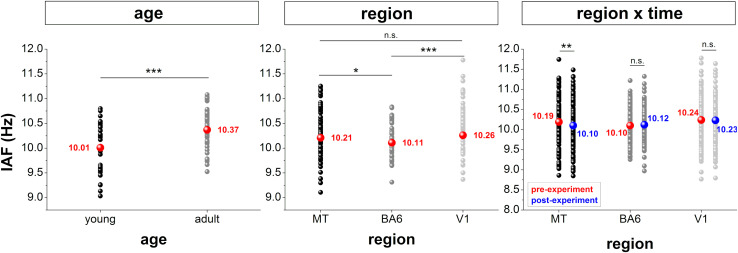
Summary of control analyses results indicating the effects of age, cortical region, and time on IAF. Figures reflect the mean (colored dot) as well as the values for all participants included (black/gray dots). n.s. = not significant, *p < 0.05, **p < 0.01, and ***p < 0.001.

Multiple regression analyses revealed a significant influence of age on the IAF in all three cortical areas (MT: t_110_ = 2.19, *p* = 0.031; BA6: t_109_ = 3.10, *p* = 0.002; V1: t_110_ = 3.12, *p* = 0.002). The ANOVA comparing participants corresponding to the highest and lowest quartile yielded a significant main effect for AGE (*F*_1,83_ = 14.77, *p* < 0.001, η_*p*_^2^ = 0.15) and REGION (*F*_2,166_ = 7.13, *p* = 0.001, η_*p*_^2^ = 0.08) indicating a higher IAF for older (20.2 ± 3.2) when compared to younger (12.3 ± 0.7) participants as well as in the two visual regions when compared to the motor region (MT vs. BA6: *p* = 0.025, MT vs. V1: *p* = 0.516, V1 vs. BA6: *p* < 0.001). A significant AGE × REGION (*F*_2,166_ = 4.47, *p* = 0.013, η_*p*_^2^ = 0.05) effect suggested that age dependent differences across regions were more pronounced in older when compared to younger participants. Results from control analyses are presented in Figure 2.

Daytime, training experience, and training load were not identified as predictors in the multiple regression model. Similarly, comparisons between quartiles did not reveal significant main effects for GROUP (daytime: *F*_1,71_ = 1.27, *p* = 0.263, η_*p*_^2^ = 0.018; training experience: *F*_1,64_ = 0.01, *p* = 0.909, η_*p*_^2^ < 0.001; training load: *F*_1,57_ = 1.05, *p* = 0.310, η_*p*_^2^ = 0.018), or a GROUP X TIME interaction (daytime: *F*_2,142_ = 1.75, *p* = 0.178, η_*p*_^2^ = 0.023; training experience: *F*_2,128_ = 1.98, *p* = 0.142, η_*p*_^2^ = 0.03; training load: *F*_2,114_ = 0.01, *p* = 0.993, η_*p*_^2^ < 0.001) for any of the parameters. However, all comparisons confirmed the region effect, indicating a lower IAF in the motor region when compared to the visual regions (*p* < 0.047).

Comparing the male and female participants in an ANOVA did not reveal a significant effect for GENDER (*F*_1,143_ = 0.23, *p* = 0.629, η_*p*_^2^ = 0.002) or a GENDER X REGION interaction (*F*_2,286_ = 1.91, *p* = 0.150, η_*p*_^2^ = 0.01). A main effect for REGION (*F*_2,286_ = 9.69, *p* < 0.001, η_*p*_^2^ = 0.06) confirmed a lower IAF in BA6 when compared to both visual regions (*p* < 0.012).

### Group Comparisons

**FIGURE 3:**
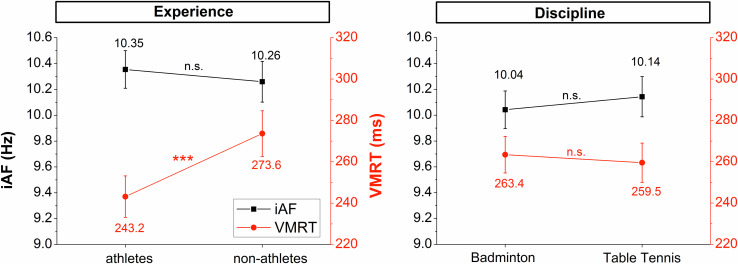
Group comparisons of IAF (black lines) and VMRT (red/gray) lines for the parameter “experience” (athletes vs. non-athletes) and “discipline” (badminton players vs. table tennis players. Error bars reflect 95% confidence intervals. n.s. = not significant, ***p < 0.001.

The ANCOVA analysis addressing differences in the IAF based on the performance level by comparing adult badminton athletes and non-athletes did not yield significant effects for the factor GROUP (*F*_1,56_ = 0.88, *p* = 0.352, η_*p*_^2^ = 0.02) or a GROUP × REGION (*F*_2,112_ = 0.40, *p* = 0.625, η_*p*_^2^ = 0.03) interaction. This pattern of result was also observed for the GROUP (*F*_1.80_ = 1.522, *p* = 0.221, η_*p*_^2^ = 0.02) and GROUP X REGION (*F*_2,160_ = 0.01, *p* = 0.963, η_*p*_^2^ < 0.001) effects when comparing young badminton and young table tennis athletes. Figure 3 presents the results on group comparisons.

### Multiple Regression and Partial Correlation Analyses

Multiple regression analyses to predict the IAF in the visual and motor regions revealed a significant model for the IAF in area MT (*F*_7,120_ = 3.15, *p* = 0.004, *r* = 0.39). However, the adjusted *r*^2^ revealed 11% explained variance and only the control variable reflecting the pre–post change in IAF did significantly contribute to the model (*p* < 0.001). All behavioral and neurophysiological variables did not reach significance (*p* > 0.265). The same pattern of result was observed in the primary visual cortex. While the regression model reached significance (*F*_7,120_ = 3.93, *p* = 0.001, *r* = 0.43), 14% of variance were explained and only the control variable of pre–post change in the IAF significantly contributed to the model (*p* < 0.001) while behavioral and neural parameters failed the significance threshold (*p* > 0.066). The model for predicting the IAF in motor areas did not reach the significance level (*F*_7,120_ = 1.76, *p* = 0.101, *r* = 0.305) and only explained 4% of the variance. The statistical metrics of the multiple regression analyses are summarized in Table 3.

**TABLE 3:** Results of the multiple regression analysis predicting the IAF in the three cortical regions of interest.

Predictor	Primary visual area (V1) *R*^2^ = 0.139, *p* = 0.001			Visual motion area (MT) *R*^2^ = 0.106, *p* = 0.004			Motor region (BA6) *R*^2^ = 0.040, *p* = 0.101		
	Std. Beta	*t*	*p*-level	Std. Beta	*t*	*p*-level	Std. Beta	*t*	*p*-level
EMG onset	–0.41	–1.30	0.195	–0.25	–0.80	0.427	–0.27	–0.83	0.409
VMRT	0.50	1.86	0.066	0.24	0.88	0.381	0.41	1.45	0.149
N2 latency	–0.13	–1.22	0.223	–0.12	–1.11	0.265	–0.13	–1.23	0.215
N2-r latency	0.06	0.40	0.690	–0.01	–0.06	0.950	–0.09	–0.06	0.955
BA6 negativity latency	–0.06	–0.69	0.495	–0.01	–0.07	0.948	–0.06	–0.61	0.542
Age	0.09	0.87	0.384	–0.01	–0.06	0.954	0.62	0.60	0.549
**ΔIAF (pre-post)**	**−0.32**	**−3.77**	** < 0.001**	**−0.37**	**−4.23**	** < 0.001**	**−0.192**	**−2.15**	**0.034**

*Std. Beta = standardized beta coefficient. R^2^ values reflect the adjusted R. Bold values indicate significant contribution to the regression model.*

**FIGURE 4:**
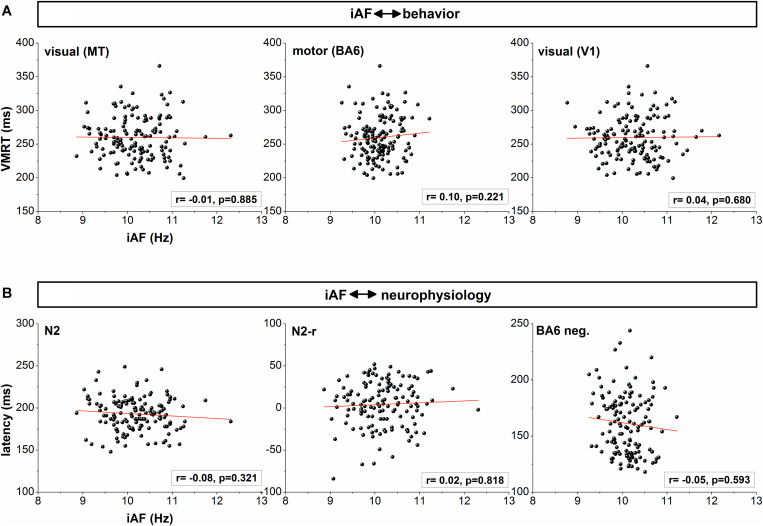
**(A)** correlation between IAF in visual (MT, V1) and motor [pre- and supplementary motor cortex (BA6)] regions and reaction speed (VMRT). **(B)** correlation between IAF and latency of event-related potentials corresponding to the motion sensitive visual area MT (N2, N2-r) and BA6 (BA6 negativity). For correlations with the N2 and N2-r latency, IAF was determined for electrode positions corresponding to area MT. For BA6 negativity correlations, IAF was calculated in electrodes related to BA6.

These findings were confirmed by the partial correlation analyses correcting for the influence of age and pre–post changes of the IAF in area MT. All analyses did not indicate any systematic relation between the IAF and behavioral performance as reflected by EMG onset (MT: t_145_ = −0.15, *r* = −0.01, *p* = 0.878; BA6: t_145_ = 0.95, *r* = 0.08, *p* = 0.346; V1: t_145_ = −0.25, *r* = −0.02, *p* = 0.802) and VMRT (MT: t_145_ = −0.12, *r* = −0.01, *p* = 0.885; BA6: t_145_ = 0.12, *r* = 0.10, *p* = 0.221; V1: t_145_ = 0.41, *r* = 0.04, *p* = 0.680). Similarly, no correlations were observed between the IAF in area MT and N2 (t_143_ = −0.99, *r* = −0.08, *p* = 0.321) or N2-r latency (t_134_ = 0.23, *r* = 0.02, *p* = 0.818) as well as between the IAF in motor areas and the BA6 negativity latency (t_136_ = −0.53, *r* = −0.05, *p* = 0.593). Correlation results are presented in Figure 4.

## Discussion

While several experiments reported a direct relation between the individual alpha frequency (IAF), cognitive performance, and visual perception, reaction tasks and underlying neural processes remained largely unconsidered. This study investigated the relation between the IAF and visuomotor reaction speed as well as the IAF and neural correlates of visual perception and processing. IAF was calculated in visual and motor areas in a large group of athletes from different sports as well as non-athletes. In contrast to the hypotheses, there was no statistically significant effect of sport experience or sport discipline on the IAF. Similarly, no relation between the IAF and any of the behavioral or neurophysiological performance parameters was observed in the partial correlation and multiple regression analyses. In line with this, supplementary analyses did not reveal any significant differences in the IAF between groups classified based on visuomotor reaction time or neural activation speed. These findings suggest that while the IAF may be associated with cognitive abilities and visual discrimination, it is not related to basic perceptual-motor performance. Accordingly, while a higher IAF may increase the *amount* and *temporal resolution* of thalamo-cortical and cortical information transfer, the *speed* of visual signal transmission seemed to be unaffected.

### IAF and Control Analyses

The individual alpha frequency has previously been shown to vary with age (Klimesch, 1999). Up to an age of about 20 years, the individual alpha frequency increases (Somsen et al., 1997; Richard Clark et al., 2004; Cragg et al., 2011) while a decline was observed after an age of 40 (Berchicci et al., 2011; Scally et al., 2018). These findings were confirmed in the control analyses revealing a higher IAF in older (20.2 years) when compared to younger participants (12.3 years). Further, a positive relation between age and IAF across all three cortical regions of interest support an increase in the individual alpha frequency with age. However, with a maximum correlation coefficient across all three regions of interest (electrodes representing V1, MT, BA6) of *r* = 0.29 corresponding to an explained variance of only about 8%, the relation between age and IAF is weak. Nonetheless, the findings of this study add further support to previous research suggesting that maturation is paralleled by an increase in individual alpha frequency.

In this study, regions for the IAF assessment were defined based on previous research showing a direct relation between cortical activity in visual and motor areas and visuomotor reaction performance (Hülsdünker et al., 2016, 2017b, 2019). It was observed that the fronto-central IAF corresponding to the pre-and supplementary motor cortex was significantly lower when compared to the two visual regions. This pattern of result is well in accordance with the previous research by Chiang et al. (2011) likewise indicating lower fronto-central alpha mu-rhythms when compared to parietal or occipital individual alpha frequencies.

Interestingly, changes in the IAF between the pre- and the post-test were observed only for cortical areas corresponding to area MT. These findings indicate focal changes in the peak alpha frequency dependent on the task demands. In this experiment, most visual stimulations (70–90%) addressed the visual motion system as represented by area MT. The reduction in peak alpha frequency may thus represent fatigue effects induced after long-term engagement in perception and visuomotor reaction tasks. In fact, a slowing of cortical peak frequency has previously been suggested to be associated with fatigue following cognitive (Barwick et al., 2012; Trejo et al., 2015) and motor tasks (Ng and Raveendran, 2007). Further, Ng and Raveendran (2007) reported that reductions in the peak alpha frequency after an exhaustive hand grip exercise were most pronounced in the cortical motor regions. Although the authors only investigated eight participants in their study, the results support the assumption that selective IAF reductions observed in higher visual areas (area MT), but not primary visual regions (V1) may be attributable to the strong engagement of visual motion regions in the experimental tasks.

In sum, the control analyses confirmed the previously reported age-dependence of alpha peak frequencies as well as the difference between fronto-central and parieto-occipital rhythms. Furthermore, the selective IAF decrease over area MT following the experiment suggest spatially focal modulations of individual alpha frequency dependent on the task demands.

### IAF and Visuomotor Performance

Across group comparisons, regression and correlation analyses, no statistically significant relation between IAF and visuomotor performance was observed. Although athletes have previously been shown to outperform non-athletes in visuomotor reaction speed, there was no group difference in IAF. Similarly, the IAF was neither correlated to any visuomotor performance (EMG onset, VMRT) or neural activation (N2, N2-r, and BA6 negativity latency) parameter, nor was it predicted in the multiple regression model suggesting that the resting-state IAF is not related to the speed of visuomotor processes both on the neural and behavioral level. The supplementary classification comparisons confirmed these findings.

The combined pattern of results contrasts our hypotheses and several experiments in the cognitive domain suggesting either differences in the peak alpha frequency between high and low performers or a direct relation between behavior and IAF. In a working memory experiment, Richard Clark et al. (2004) reported that peak alpha frequency together with age explained 10.2% of the variance in a reverse digit span test. Similarly, Grandy et al. (2013) showed a correlation between the individual alpha frequency and cognitive performance scores. Further research revealed a correlation between IAF and hippocampal volume (Garcés et al., 2013), a brain structure well established to be essential for memory functions (Bird and Burgess, 2008). Consequently, impaired cognitive abilities associated with neurological disorders such as traumatic brain injury (Angelakis et al., 2004), mild cognitive impairment (Garcés et al., 2013), or autism spectrum disorder (Dickinson et al., 2018) were accompanied by a lower individual alpha frequencies.

In addition to cognitive processes, the frequency of alpha oscillations also affects visual perception. Samaha and Postle (2015) identified a negative relation between the IAF and the two-flash fusion threshold in a visual stimulation experiment. Participants exhibiting a higher resting state and pre-stimulus IAF were characterized by a lower two-flash fusion threshold. In other words, a higher IAF allowed participants to differentiate between separate light flashes at lower interstimulus intervals when compared to participants with a lower IAF who perceived the two light flashes as one. The authors interpreted these findings as reflecting a higher temporal resolution associated with a higher IAF. This interpretation is further supported by the study of Cecere et al. (2015) investigating the time window of sound-induced double flash illusions. In line with temporal framing of information based on the alpha rhythm, the authors reported a direct relation between the IAF and the temporal width of the illusion window. Entrainment of cortical rhythms at different frequencies around the IAF using tACS over visual areas confirmed these findings.

The concept of perceptual cycles (VanRullen, 2016) provides a conceptual framework for these findings. Information transmission is suggested to be framed by the rhythmic fluctuation of excitatory and inhibitory states in the alpha frequency affecting the probability of neural signal generation and thus information transfer. Experimentally, this assumption is supported by previous research investigating cortical and sub-cortical generators of the alpha rhythm and its effect on information processing. Single-cell recordings in the thalamus revealed an alpha phase-dependent thalamo-cortical information transfer (Lorincz et al., 2009) that is well in accordance with the concept of neural pacemaker cells in the visual system (Bollimunta et al., 2008) and models on cortical information processing such as the inhibition timing hypothesis (Wolfgang Klimesch, 2012). A higher IAF accompanied by a higher rate of excitation-inhibition cycles would provide a greater information transfer per time which may facilitate cognitive processes by a higher rate of information transfer as well as visual perception by improving the temporal resolution of visual information integration.

Based on the abovementioned findings, the missing relation between the IAF and visual perception and visuomotor reaction may be surprising. Specifically, following the threshold model of visual motion perception proposed by Stevens et al. (2009), it was hypothesized that a higher rate of information transfer at a higher IAF may accelerate motion information integration and thus cortical activation and visuomotor reaction time.

One possible explanation for the results in this study may be related to the different behavior of the IAF in visual integration and segregation tasks. Previous research performed by Samaha and Postle (2015) or Cecere et al. (2015) reported a positive effect of a higher IAF on the segregation of visual and audio-visual information. However, research by Wutz et al. (2018) suggests that the IAF behaves differently dependent on the visual task demands. Specifically, the IAF was higher for a segregation when compared to an integration task. Accordingly, a performance determining role of the IAF may only be expected for segregation tasks that require the differentiation between individual visual stimuli.

In contrast, since the visuomotor reaction task is characterized by the perception of a motion onset during a 200 ms stimulation, this may be best defined as an integration task. Following the threshold model of Stevens et al. (2009), visual motion information would be continuously integrated until a threshold is reached that identifies the visual stimulus as a visual motion and initiates the motor response. Following this assumption, the IAF should not affect the reaction speed which is well in line with the findings of this study. Specifically, from an integration point of view, it would not make a difference if more motion information were transferred in less cycles (low IAF) or vice versa (high IAF). The overall amount of motion information would be similar.

Another line of argument supporting the findings of this study may assume that while a higher IAF improves the *amount* and *temporal resolution* of information transfer per unit of time and thus allow higher computational capacity and discrimination of visual information, it would not affect the *speed* of perceiving a single stimulus. Considering the alpha-phase dependence of visual information transmission on the thalamic and cortical level (Bollimunta et al., 2008; Lorincz et al., 2009), the speed may depend on the phase at visual signal arrival. If a single stimulus is presented (i.e., motion onset in this experiment), the probability to arrive at an excitatory (fast information transfer) or inhibitory (slow information transfer) does not depend on the alpha frequency since the proportion of inhibitory and excitatory phases is always 50%. These considerations are in line with the concept of perceptual cycles (VanRullen, 2016) indicating that visual perception speed may depend more on the pre-stimulus alpha phase rather than the resting-state alpha frequency. This is also supported by the modulations of VEP latency dependent on the pre-stimulus phase in the IAF (Hülsdünker et al., 2018b). Future studies should investigate if the pre-stimulus IAF phase affects the speed of visuomotor reactions.

Importantly, although Jin et al. (2006) reported a direct relation between resting-state individual alpha frequency and reaction time, this was only observed for a complex conflict reaction task. In contrast, there was no relation to a simple reaction time test suggesting the IAF is related to a more complex cognitive rather than simple perceptual-motor processes. Furthermore, the results are in accordance with a recent study of Christie et al. (2017) likewise observing no performance-related differences in IAF between high and low performers in a sport-specific motor task. The authors discussed that the resting state individual alpha frequency may only be related to cognitive factors which is supported by the results of this study. Additional support to this hypothesis is provided by the missing relation between IAF and visual as well as visuomotor processes on the neurophysiological level. IAF was neither correlated to the latency of visual evoked N2 or N2-r components over area MT nor the BA6 negativity potential corresponding to pre- and supplementary motor areas. Similarly, group comparisons between participants with faster visual (N2, N2-r latency) and motor (BA6 negativity latency) activation did not reveal differences in IAF.

### Limitations

This study provides a comprehensive analysis of the effects of IAF on neural information processing and behavioral reaction performance in different groups of athletes and non-athletes. However, due to the experimental design of previous research, groups were different in sample size which may have affected the statistical outcome at least for the group and performance comparisons. Furthermore, it needs to be considered that while an adult control group was available, young non-athletes as a reference for the young badminton table tennis athletes as well as a group of adult high level table tennis athletes was not included.

Furthermore, this study focused on the relation between resting-state IAF and visuomotor performance on the neural and behavioral level. While this approach was based on a series of research in the cognitive domain, it remains unclear if the IAF *during* the task may be a better predictor of task performance. Since the IAF changes dependent on perceptual (Wutz et al., 2018), cognitive (Haegens et al., 2014), or motor (Hülsdünker et al., 2015) task demands and was also affected after the experiment in this study, in-task IAF measures should be addressed in future research.

## Conclusion

This study investigated the relation between the resting-state individual alpha frequency (IAF), neural activation speed in visual and motor regions, and simple visuomotor reaction performance in a large sample of high-level athletes and non-athletes. Results confirmed an age-dependence of the individual alpha frequency as well as lower IAF values in motor when compared to visual regions. However, IAF was neither related to the visuomotor reaction speed nor cortical correlates of visual (N2, N2-r) and motor (BA6 negativity) processes. The results contrast previous studies on cognitive performance and visual discrimination and indicate that a higher IAF may improve the amount and temporal resolution but not the speed of information transfer.

## Data Availability Statement

The data analyzed in this study is subject to the following licenses/restrictions: The original datasets will be only made available on request. Although personal data (e.g., name or address) of the participants are not included in the datasets, the risk of identifying athletes based on their age, gender and sport is comparatively high since the groups of highly trained participants is small in badminton and table tennis. This especially applies to the groups of young top-level athletes who are under legal age. Requests to access these datasets should be directed to TH (thorben.huelsduenker@lunex-university.net).

## Ethics Statement

The studies involving human participants were reviewed and approved by Research Ethics Committee, German Sport University Cologne in accordance with the declaration of Helsinki. Written informed consent was provided by all participants. For athletes under legal age, the consent form was also signed by the participant’s parents or guardian/next of kin.

## Author Contributions

TH designed the study, conducted the data acquisition and analysis, interpreted the findings, and wrote the manuscript. AM was involved in designing the study as well as interpreting the result and reviewing the manuscript. Both authors contributed to the article and approved the submitted version.

## Conflict of Interest

The authors declare that the research was conducted in the absence of any commercial or financial relationships that could be construed as a potential conflict of interest.

## Supplementary Material

Supplementary Figure 1 Results of classification comparisons (fast vs. medium vs. slow) for IAF (black line) and VMRT (red/gray line). Classifications are based on behavioral (VMRT) or neurophysiological parameters (N2, N2-r, BA6 negativity latency). Since ANOVA results did not yield a group × region interaction, IAF values reflect the average across all cortical areas of interest. Error bars reflect 95% confidence intervals. n.s. = not significant, ***p* < 0.01, ****p* < 0.001.

Supplementary Table 1 Group characteristics based on performance classification. Data represent mean (±standard deviation).
