# Supplementary material for: Visual Perception and Visuomotor Reaction Speed Are Independent of the Individual Alpha Frequency
Source: Front Neurosci. 2021 Apr 8;15:620266. doi: 10.3389/fnins.2021.620266 (PMC8060564; doi:10.3389/fnins.2021.620266)
Supplement: Supplementary Figure 1 — Results of classification comparisons (fast vs. medium vs. slow) for IAF (black line) and VMRT (red/gray line). Classifications are based on behavioral (VMRT) or neurophysiological parameters (N2, N2-r, BA6 negativity latency). Since ANOVA results did not yield a group × region interaction, IAF values reflect the average across all cortical areas of interest. Error bars reflect 95% confidence intervals. n.s. = not significant, **p < 0.01, ***p < 0.001. [file Data_Sheet_1.PDF]

Supplementary methods

Performance classification

To test the hypotheses if fast-reacting athletes may be characterized by a higher IAF, we used a classification approach to compare the IAF of fast and slow responders. The group of participants was subdivided into terciles based on their EMG onset latency and visuomotor reaction time (VMRT). The fastest 33% of participants were assigned to the high performance group while the middle 33% and bottom 33% were assigned to the medium performance and low performance group, respectively. An ANCOVA with the within-subject factor GROUP (high performers, medium performers, low performers), the within-subject factor REGION (MT, V1, BA6) and age and pre-post IAF change score in MT as covariates evaluated the effects of reaction performance on IAF. Since the IAF may further be associated with the speed of neural activation, the same analyses were performed after classifying the participants based on their N2, N2-r and BA6 negativity latency indicating activation speed in visual and motor regions of the cortex. Average behavioral (EMG onset/VMRT) and neurophysiological (N2/N2-r/BA6 negativity) data for all terciles are summarized in supplementary Table 1.

To confirm differences in EMG onset and VMRT in the group comparison and classification approach, ANCOVA analyses described above for IAF were repeated with EMG onset and VMRT as the dependent variables, respectively. Results revealed highly significant ($p<0.001$) effects except for the discipline comparison (young badminton vs. young table tennis) as well as when classification was based on the BA6 negativity latency. Since these tests were only for control and confirmation purposes, they are not included in the results section.

Supplementary results

Performance classification

No GROUP or GROUP X REGION effects on IAF were observed when classification was based on EMG onset (GROUP: $F_{2,139}=0.716$, $p=0.491$, $\eta_p^2=0.01$; GROUP X REGION: $F_{4,278}=2.003$, $p=0.114$, $\eta_p^2=0.01$) or VMRT (GROUP: $F_{2,139}=0.538$, $p=0.585$, $\eta_p^2=0.08$; GROUP X REGION: $F_{4,278}=0.979$, $p=0.404$, $\eta_p^2=0.01$).

Similar to the behavioral results, classifying participants based on N2 and N2-r latency in the visual system did not indicate a GROUP (N2: $F_{2,135}=1.873$, $p=0.158$, $\eta_p^2=0.027$; N2-r: $F_{2,126}=0.04$, $p=0.958$, $\eta_p^2<0.001$) or GROUP X REGION interaction effect for IAF (N2: $F_{2,135}=0.868$, $p=0.458$, $\eta_p^2=0.013$; N2-r: $F_{4,252}=0.86$, $p=0.662$, $\eta_p^2=0.009$). The same applied to the GROUP ($F_{2,135}=0.30$, $p=0.739$, $\eta_p^2=0.005$) and GROUP X REGION ($_{4,258}=0.39$, $p=0.816$, $\eta_p^2=0.006$) results for the classification based on BA6 negativity latency. Findings on group comparisons are illustrated in supplementary Figure 1.
