# Supplementary material for: Visual Perception and Visuomotor Reaction Speed Are Independent of the Individual Alpha Frequency
Source: Front Neurosci. 2021 Apr 8;15:620266. doi: 10.3389/fnins.2021.620266 (PMC8060564; doi:10.3389/fnins.2021.620266)
Supplement: Supplementary Table 1 — Group characteristics based on performance classification. Data represent mean (±standard deviation). [file Table_1.docx]

Supplementary Table 1: Group characteristics based on performance classification. Data represent mean (±standard deviation)

| **Classification Parameter** | **Fast (ms)** | **Medium (ms)** | **Slow (ms)** |
| --- | --- | --- | --- |
| EMG onset | 171.1(±15.5) | 201.8(±7.0) | 238.3(±18.3) |
| VMRT | 227.4(±12.3) | 256.3(±8.2) | 294.3(±20.2) |
| N2 latency | 171.4(±10.1) | 193.0(±5.3) | 213.5(±11.4) |
| N2-r latency | 32.1(±9.1) | 5.8(±6.0) | -25.0(±17.7) |
| BA6 negativity latency | 132.0(±6.1) | 159.0(±9.6) | 191.9(±18.4) |
