# Supplementary figures and images for: Visual Perception and Visuomotor Reaction Speed Are Independent of the Individual Alpha Frequency
Source: Front Neurosci. 2021 Apr 8;15:620266. doi: 10.3389/fnins.2021.620266 (PMC8060564; doi:10.3389/fnins.2021.620266)

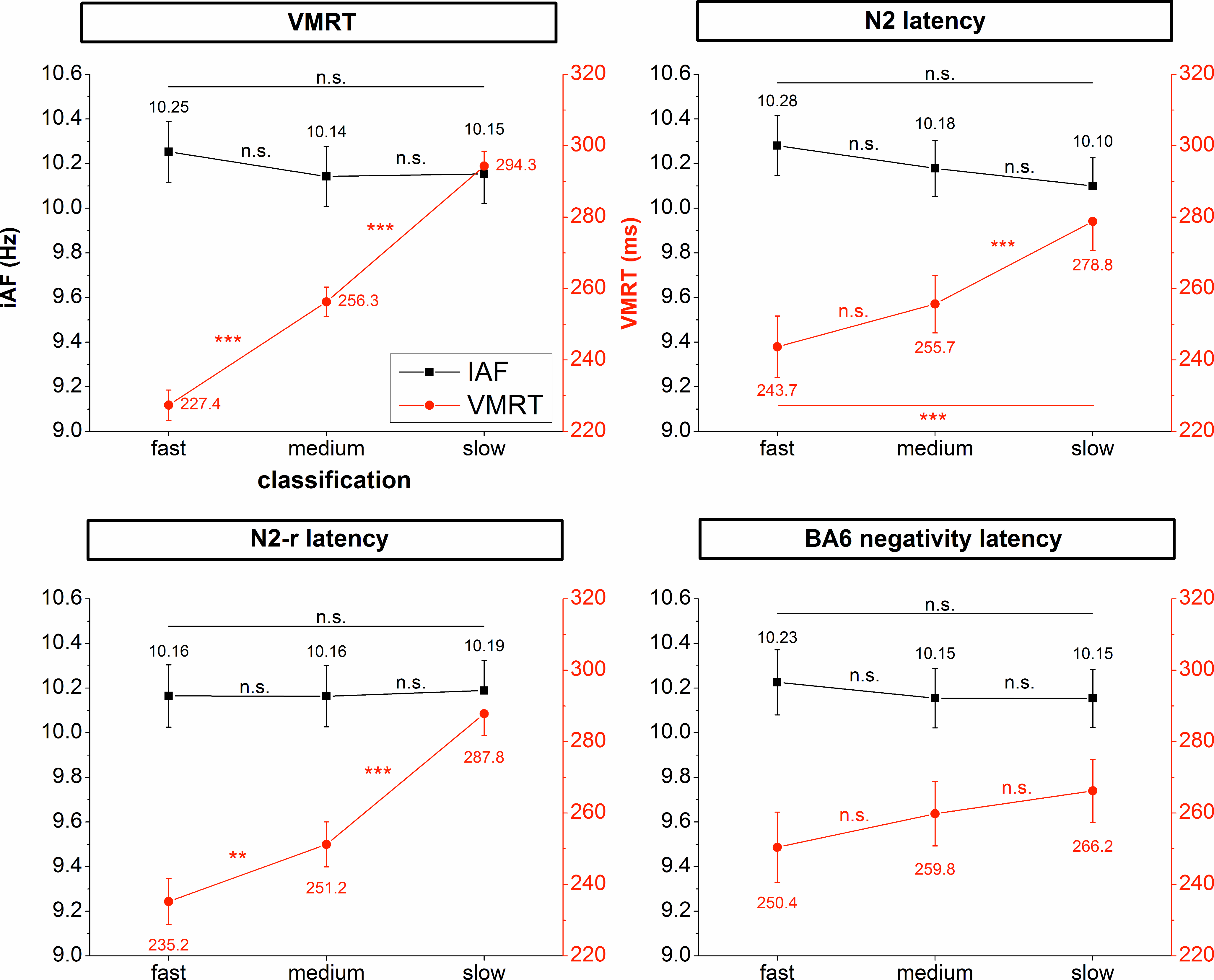

Supplement: Supplementary file 3 [file Image_1.TIF]
